# Discovery of Tumor Markers for Gastric Cancer by Proteomics

**DOI:** 10.1371/journal.pone.0084158

**Published:** 2014-01-03

**Authors:** Jeng-Yih Wu, Chun-Chia Cheng, Jaw-Yuan Wang, Deng-Chyang Wu, Jan-Sing Hsieh, Shui-Cheng Lee, Wen-Ming Wang

**Affiliations:** 1 Department of Internal Medicine, Kaohsiung Municipal Ta-Tung Hospital, Kaohsiung, Taiwan; 2 Division of Gastroenterology, Department of Internal Medicine, Kaohsiung Medical University Hospital, Kaohsiung, Taiwan; 3 Department of Medicine, Faculty of Medicine, College of Medicine, Kaohsiung Medical University, Kaohsiung, Taiwan; 4 Institute of Nuclear Energy Research, Atomic Energy Council, Executive Yuan, Taiwan; 5 Department of Surgery, Kaohsiung Medical University Hospital, Kaohsiung,Taiwan; 6 Department of Internal Medicine, Kaohsiung Municipal Hsiao-Kang Hospital, Kaohsiung Medical University, Kaohsiung, Taiwan; Duke University Medical Center, United States of America

## Abstract

Gastric cancer (GC) has a high rate of morbidity and mortality among various cancers worldwide. The development of noninvasive diagnostic methods or technologies for tracking the occurrence of GC is urgent, and searching reliable biomarkers is considered.This study intended to directly discover differential biomarkers from GC tissues by two-dimension-differential gel electrophoresis (2D-DIGE), and further validate protein expression by western blotting (WB) and immunohistochemistry (IHC).Pairs of GC tissues (gastric cancer tissues and the adjacent normal tissues) obtained from surgery was investigated for 2D-DIEG.Five proteins wereconfirmed by WB and IHC, including glucose-regulated protein 78 (GRP78), glutathione s-transferase pi (GSTpi), apolipoprotein AI (ApoAI), alpha-1 antitrypsin (A1AT) and gastrokine-1 (GKN-1). Among the results, GRP78, GSTpi and A1ATwere significantlyup-regulated and down-regulated respectively in gastric cancer patients. Moreover, GRP78 and ApoAI were correlated with A1AT for protein expressions.This study presumes these proteins could be candidates of reliable biomarkers for gastric cancer.

## Introduction

Although theprevalence of gastric cancer is declining and varying geographically, it remains one of the most common cancers in Asian countries and is the fourth most commonly occurring cancer (9% of all cancers) worldwide. The age-standardized incidence rates (ASR) are greater than 20 per 100,000 in high risk countries such as China, Japan and Korea[1] [2], [3]. It is also the second leading cause of cancer death in both sexes worldwide (737,000 deaths, 9.7% of the total). In Eastern Asia, the mortality rates have been estimated as about 28.1 per 100,000 in men and 13.0 per 100,000 in women, whilein Northern America, they are about 2.8 and 1.5 respectively[4].

Five-year survival rates have ranged from 90% to less than 5 percent, mainly depending on the stage of diagnosis[5], [6].If gastric cancer can be detected and treated in early stages, the five-year survival rateis better than 90%; however, there isno apparent or specific symptom in early-stage gastric cancer.Thus, early detectionof gastric cancer becomesmore difficult.Although serum pepsinogen (PG) testssuch as low PGI concentration and/or low PGI/II ratio were suggestive screening tests in high-risk countries such as Japan, they weregood indicators of atrophic gastritis rather than diagnostic markersof gastric cancer[7]–[9].Essentially, endoscopy has beenthe promising tool with 2.7 to 4.6-times higher detection rate than barium studies[10]. However,early gastric cancer diagnosis by endoscopydependson professional skill.

Some non-invasive biomarkers for diagnosis or follow-up in gastrointestinal and hepatic tumorshave been reported. For example, alpha-fetoprotein (AFP) is one of the clinically useful biomarkers for the diagnosis and follow-up of hepatocellular carcinoma (HCC).AFP was elevated above 20 ng/mL in one study in more than 70% of patients with HCC[11]. According to the conclusions of the Barcelona-2000 European Association for the Study of the Liver (EASL) conference, HCC could be diagnosed without biopsyin cases where AFP is greater than 400 ng/ml with a nodule larger than 2 cm, showing evidence of arterial hypervascularization in cirrhotic patients[12].Incolorectal carcinoma(CRC),preoperative carcinoembryonic antigen (CEA) level is a highly significant prognostic covariate and it is recommended by the American Society of Clinical Oncology (ASCO) that it should be tested pre-operatively to provide prognostic information [13]. Serial elevations of CEA indicate higher possibility of recurrence of CRC. However, there is no reliable biomarker for gastric cancer.

Therefore, searching for reliable biomarkers for gastric cancer is very important for clinical practice. This study aimed to discover reliable protein biomarkers from matched tissues (tumor and adjacent normal tissues) by two-dimension-difference gel electrophoresis (2D-DIGE), and identify the proteins by matrix-assisted laser desorption/ionization-imaging mass spectrometry (MALDI-IMS).

## Materials and Methods

### Tissue samples

Tissue samples were obtained after inform consents were signed. Paired samplesincluding tumor and adjacent normal tissuesfrom patients were derived from endoscopic biopsies or surgery.The tumor grades were determined according to the American Joint Commission on Cancer Staging (AJCCS) system by pathologists under methylene blue staining.Clinical information of the patients was recorded, including age, gender, tumor type, invasion and survival. Additionally, the CEA concentration in serum was also measured by radioactive immunoassay in routine medical diagnosis.The individualsenrolled in the present study have given written informed consent to publish these case details.This study was approved by the Institutional Review Board of Kaohsiung Medical University Chung-Ho Memorial Hospital (KMUH-IRB-980382).

### Two dimension-difference gel electrophoresis

**Figure 1:**
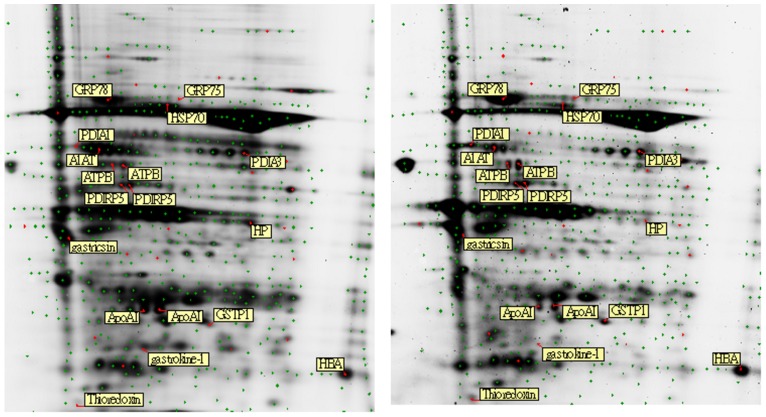
The 2D-DIGE results paired tissue samples. The differential proteins presented on gels. (left): normal; (right): cancer. Thirty-six proteins were picked by DeCyder statistical software, but only fifteen proteins were identified by PMF or PFF technique. The gel conditions: pI: 4–7; gel: 4–12%.The analytical ranges were from pI 4 to 7 forisoelectric focusing and from 4% to 12% gradient gel for sodium dodecyl sulfate- polyacrylamide gelelectrophoresis.

One (Table 1) pair of tissue samples washomogenized (PRO 200, Bertec, Taiwan) in moderate volume of lysis buffer (50 mM of Tris-HCl, 8M of urea, 4% (w/v) 3-[(3-Cholamidopropyl) dimethylammonio]-1-propanesulfonate, and pH8.5). The individual 50 µg of sample protein was labeled with 400 pmol of cy3 or cy5 separately for 30 minutes (Fig. 1B). In addition, the pooled sample mixture (100 µg)was labeled with 800 pmol of cy2 as internal standard at the same time. Subsequently the labeling reactionswere stopped by incubating 1 µL of 10 mM of lysine buffer for 30 minutes,then one-fold volume of sample buffer (8 M of urea, 20 mM of dithiothreitol, 4% (w/v) 3-[(3-Cholamidopropyl) dimethylammonio]-1-propanesulfonate, 0.5% (v/v) IPG buffer and few bromophenol blue) was added.The first separation, isoelectric focusing, was performed using cap loading onto the gel strips (7 cm, pI 4–7) at 20°C keeping under 15000voltage-hours (IPGphor system, GE Healthcare). After equilibration with sodium dodecyl sulfate, the second separationwas performed using4–12% sodium dodecyl sulfate-polyacrylamide gelelectrophoresis. The gel images were acquired (Typhoon TRIO Variable Mode Imager, GE Healthcare) using 488, 532, and 633 nm lasers with an emission filter of 520, 532, and 670 nm respectively. All images were analyzed with DeCyder 6.5 software (GE Healthcare) to select the differential proteins which wereselected depending onthe placement below a significant value of 0.05 according to Student *t*-test.

**Table 1:** Patient information of gastric cancer.

Patient	Gender	Age	Stage	Lauren's	Invasion	CEA	Survival
1	M	79	IA	ND	no	ND	ND
2	M	64	IV	ND	yes	ND	ND
3	F	49	IA	diffuse	no	3.19	yes
4	M	87	IB	intestinal	no	3.66	no
5	F	52	IV	diffuse	yes	3.15	yes
6	M	79	II	intestinal	yes	1.21	yes
7	F	74	IV	intestinal	no	0.64	yes
8	M	70	II	intestinal	no	1.28	yes
9	M	88	IIIIA	intestinal	yes	3.09	no
10	M	63	IIIIA	intestinal	no	1.1	yes
11	M	70	IA	intestinal	no	1.52	yes
12	M	75	II	intestinal	no	3.33	no

The tumor grades were determined according to the American Joint Commission on Cancer Staging (AJCCS) system. ND: non-detection.

### In-gel tryptic digestion

The gels stained with Sybro Ruby (sigma) were subsequently destained with 10% methanol and 7% acetic acid in deionized water for exactly 30 min.The visible-spots gels under a UV transilluminator (Spectroline) were used to dig for the interesting proteins manually. These gel particles were washed in 100 µL of 25 mM ammonium bicarbonate in 50% acetonitrile for 15 min, and then washed in 200 µL of 25 mM ammonium bicarbonate in deionized water for 15 min twice,and subsequently,enough acetonitrile was added to shrink the gel particles. After drying down, the individual gel particleswereincubated with 3 µL of 20 ng/µL of trypsin in 25 mM ammonium bicarbonate at 4°C for 1 hour and subsequently 3 µL of 25 mM ammonium bicarbonate was added to digest the proteins at 56°C for 1 hour. After In-gel digestion, 2 µL of 100% acetonitrile with 1% trifluoracetic acid were added to the solutions and then samples were sonicated for 10 min to release peptides from gel particles.

### Mass spectrometric analysis for protein identification

Each protein solution digested with trypsin was mixed 1∶1 with 10 mg/mlof α-cyano-4-hydroxycinnamic acid dissolved in 50% acetonitrile/0.1% trifluoracetic acid, and spotted on AnchorChip MALDI target (Bruker Daltonics GmbH, Bremen, Germany) until dry. Peptides were analyzed by MALDI-TOF/TOF UltraflexIII (Bruker Daltonics) under 20 KV with positive model, and the peak data were transferred to FlexAnalysis™ 3.0 software (Bruker Daltonics) for advanced calculation and calibration. MASCOT 2.2 (Matrix Science) was used to match the peptides with NCBI or Swiss-Prot database for protein identification. The setting was restricted to human taxonomy parameters.Meanwhile, carbamidomethyl cysteine was used as a fixed modification, and oxidized methionine as a variable modification. The probability (P) was based on mowse score calculated from −10×Log (P). Protein score greater than 56 was significant (*p*<0.05). Moreover, one of the major peptide peaks appearing on the spectrum was used to confirm the identical result by identifying the amino acid sequence, which is called peptide fragment fingerprinting method.

### Western blotting

The pairs of tissue samples were homogenized (Pro 200, Bertec) in the lysis buffer (10 mM of sodium phosphate, 0.9% sodium chloride, 1% triton-X100, pH7.4) and incubated on shaking at 4°C for 1 hour. After getting rid of the precipitated pellets by high-speed centrifugation (10000 rpm, 5 minutes), the pipetted supernatants were added to sample buffer (10 mM of sodium phosphate, 0.9% sodium chloride, 8 M of urea, 30% glycerol, 2% sodium dodecyl sulfate, 0.1% β-mercaptoethanol and 0.1% bromophenol blue) by 1∶ 1 ratio and boiled at 100°C for 5 min for protein denature.Approximately 20 µg of each sample protein were loaded onto the individual grid of 4–12% sodium dodecyl sulfate-polyacrylamide gel electrophoresis (SDS-PAGE, Invitrogen). The iblot dry blotting system (Invitrogen) was used for transforming the proteins to polyvinylidene fluoride (PVDF) membrane based on ion flowing along with a copper electrode. After using 0.5% milk to blot the PVDF membrane for 30 min, theindividual primary antibody (2 µg/ml) was added for incubation for 2 hour on shaking. The consistent secondary antibodies conjugated with horseradish peroxidase (HRP) (2 µg/ml) were incubated for 1 hour on shaking consecutively. Between the incubating processes, repeatedlywashings by PBS buffer (10 mM sodium phosphate, pH7.4 and 0.9% sodium chloride)weredone. The ECL detection system (Millipore) was performed, and the images were acquired by Imaging System (Gel Doc XR System, Bio-Rad) depending on the moderate exploring time.

### Immunohistochemistry

Immunohistochemistry was performedusing a standard peroxidase-based staining method. The tissue samples were cut by a cryostat (HM525, Microm).Fresh tissue sections (10 um) on the lysine-coated slides were driedon a heater at 37°C and consecutively immersed by sequential 75%, 95% and 100% ethanol for 1 minute for protein fixation. Briefly, endogenous peroxidase activity was quenched with 3% hydrogen peroxide for 10 minutes. The antigen episode was exposed by immersing the tissue slide in 10 mM of citrate acid within boiling water for 25 minutes. Successive incubations with the individual primary antibodyandthe HRP-conjugated secondary antibodies were performed for individual 1 hour on shaking. The AEC kit (Sigma) was used to stain the tissues, and the operation followed the attached manual. Briefly, themixed solution of 2.5 M of acetate buffer, AEC chromogenic (3-amino-9ethylcarbazole) and 3% hydrogen peroxide wasperformed instantly and consecutively incubated with tissue slides.The image staining slides were observed and acquired under a microscopy (BX51, Olympus).

### Statistics analysis

The statistic software SPSS was performed to calculate the significance according to Student *t*-test and correlation among GRP78, GSTpi, A1AT, ApoAI and GKN-1. The significantdifference (*p* value) was acceptable as less than 0.5.

## Results

### Biomarkers discovery based on 2D-DIGE

**Figure 2:**
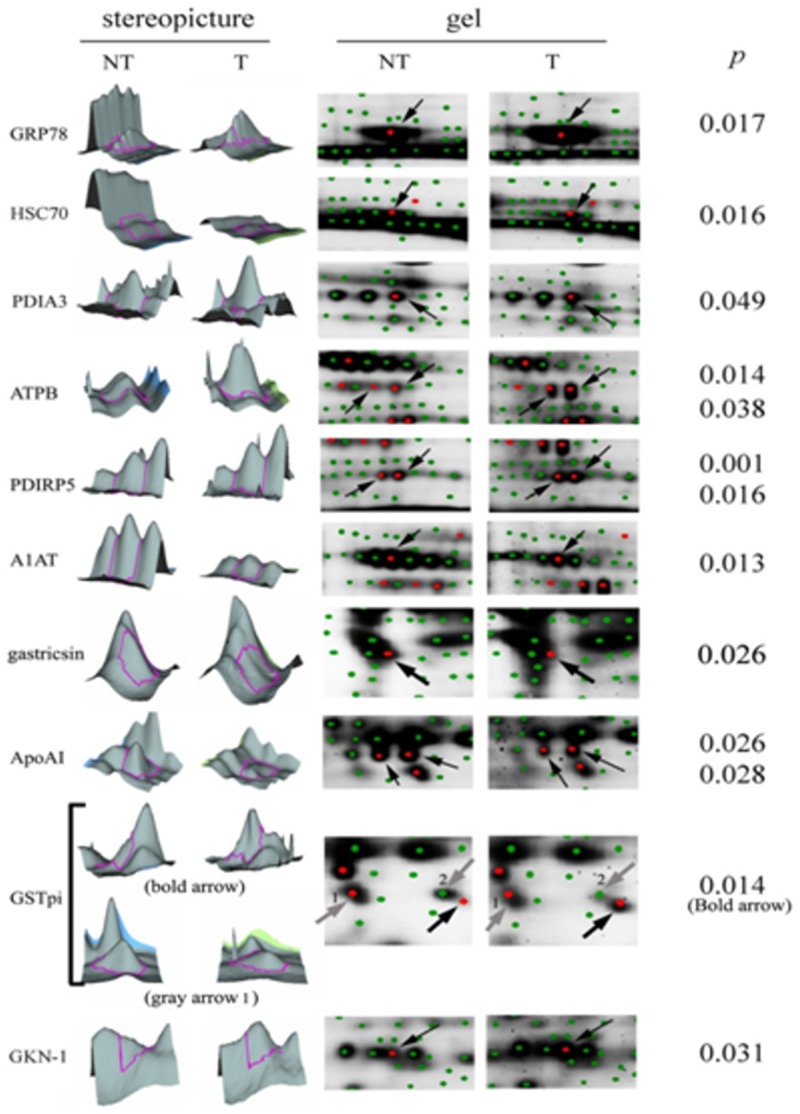
The detailed comparison between normal and tumorous tissue from 2D-DIGE. The selected putative proteins were presented as stereopictures and enlarged scales gel images. These proteins were selected according to the significant difference below 0.05 (*p*<0.05). In addition, the duplicated identification of ATPB, PDIRP5, ApoAI and GSTpi on adjacent spots indicated that they had a different modification.

Gastric cancer tissues were analyzed by 2D-DIGE and compared with paired normal tissues to search for the putative biomarkers of gastric cancer. Differences of proteinexpressions greater than 1.2 fold were putative candidates. The gel image was presentation of proteins location labeled with cy-dyes(Fig. 1). Fifteen proteins could be identified, including glucose-regulated protein 78 (GRP78), heat shock cognate 71 kDa protein (HSC70), protein disulfide-isomerase A3 (PDIA3), mitochondrial ATP synthase subunit beta (ATPB), protein disulfide isomerase-related protein 5 (PDIRP5), gastricsin, glutathione s-transferase pi (GSTpi), apolipoprotein AI (ApoAI), alpha-1 antitrypsin (A1AT) and gastrokine-1 (GKN-1).In 3 duplicated repeats, some identical proteins were found at different locations of gel and excluded under the suspicion of post-translational modification. Finally, five proteins including GRP78, GSTpi, ApoAI, A1AT and GKN-1 were confirmed and further validated as putative markers of gastric cancer. The detailed comparisons by stereopicture and enlarged gel imageswere shown in Figure 2.

### Western blotting and statistical analysis

**Figure 3:**
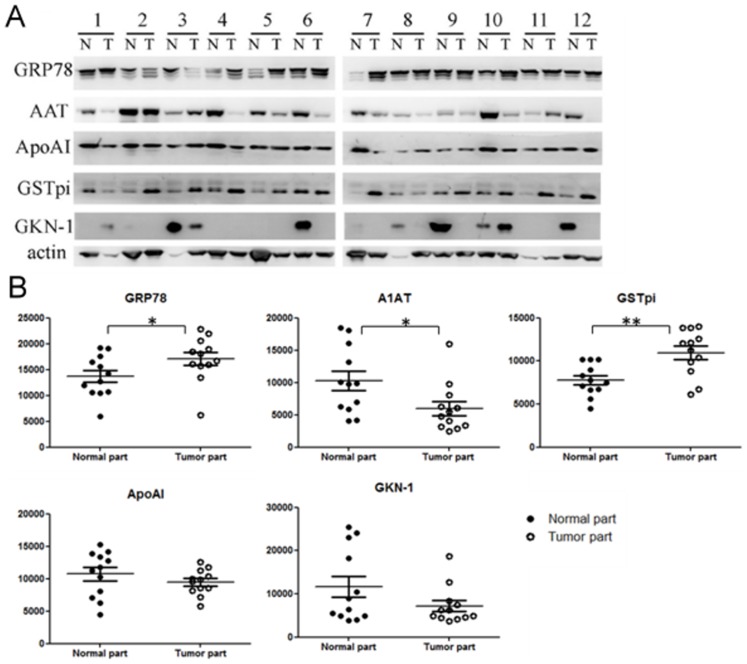
(A) By Western blotting, 12 paired of gastric cancer and normal tissues was used to validate the putative proteins including GRP78, GSTpi, A1AT, ApoAI and GKN-1. (B) The GSTpi and GRP78 were significantly over-expressed in gastric cancer tissues.Down-expressions of A1AT, ApoAI and GKN-1 were noted. **p<0.01. *p<0.05.

**Figure 4:**
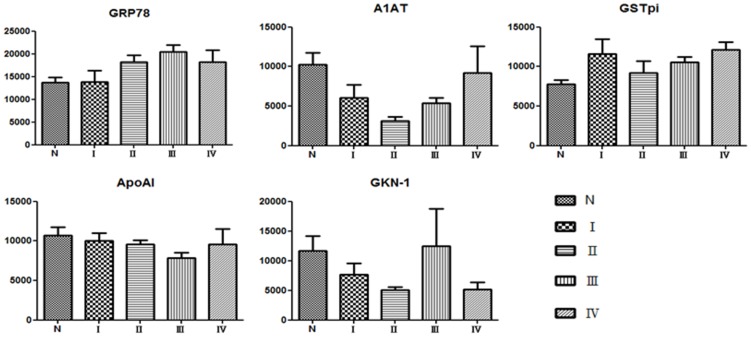
The correlations between clinical stages of gastric cancer and the expressions of proteins. Although GRP78 and GSTpi were increased in different stages of gastric cancer, no statistical significance could be found. A trend of increase with clinical stage was found in GRP78.

Twelvepairs of tissues from gastric cancer patients were used as validation group. Four patients were stage I, 3 patients were stage II, 2 patients were stage III, and 3 patients were stage IV. The clinical information was listed in Table 1.Five of the proteins (GRP78, GSTpi, ApoAI, A1AT and GKN-1) were confirmed by Western blotting.Amongthe results, GRP78 and GSTpiwere significantlyup-regulated while A1AT was significantly down-regulated in gastric cancer tissues compared to normal tissues (Fig. 3).For GSTpi, nineout of 12 patients (75%) had up-regulated protein expressions in tumortissues; on the other hand,10 of 12 patients (83%) have down-regulated A1AT protein expression.Regarding the relationship between protein expressions and clinical stages, GRP78 has a trend to increase from stage I to stage IValthough no statistical significance could be found. The expression of GSTpi and A1AT were not correlated with clinical stages (Figure 4).

**Figure 5:**
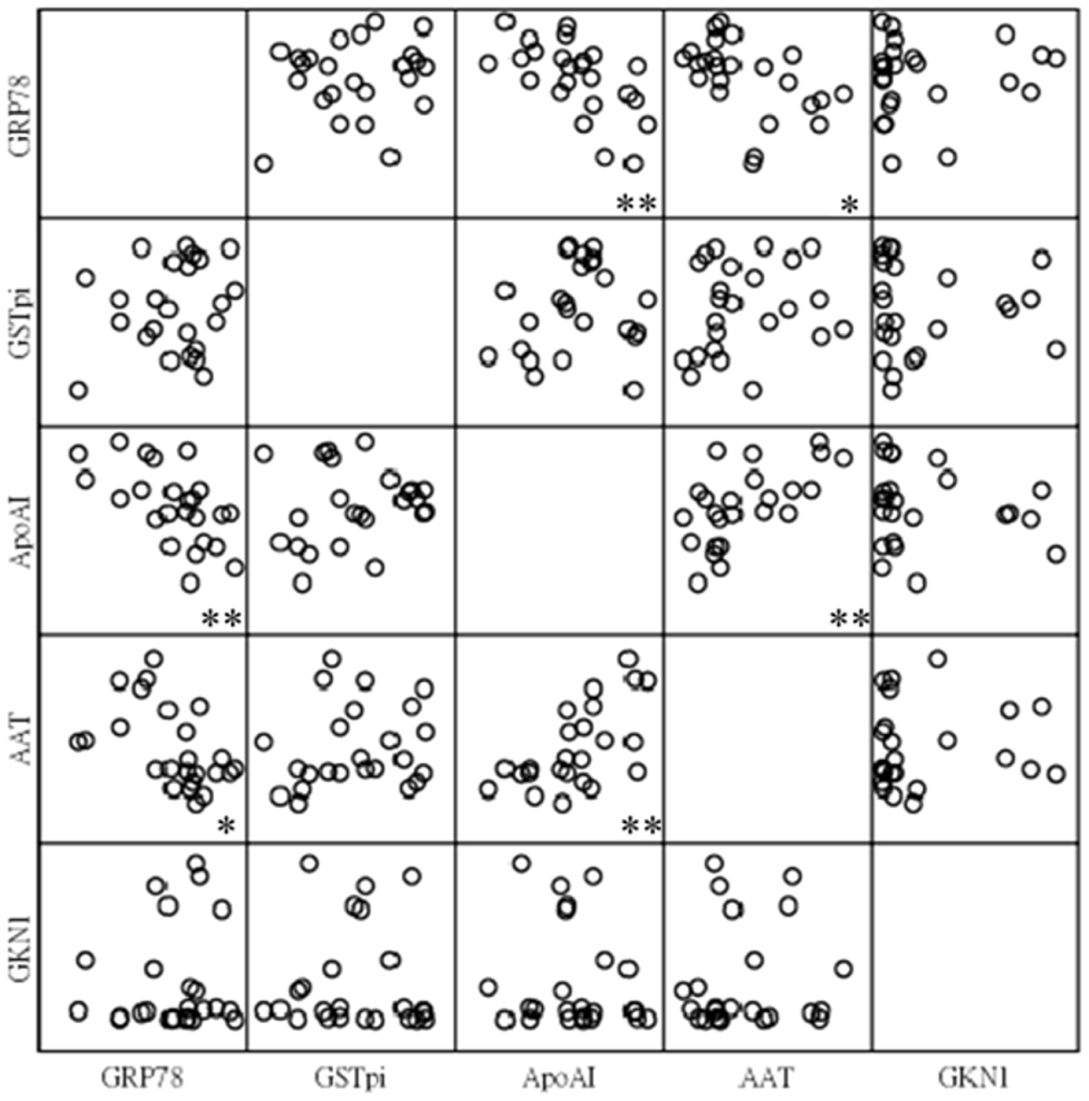
The correlation among the selected proteins. The GRP78 in these 12 pairs of tissues was expressed correlatively with that of ApoAI (r2: −0.61) and A1AT (r2: −0.49) individually. Meanwhile, ApoAI was expressed correlatively with that of A1AT (r2: 0.62). However, GSTpi and GKN-1 seemed to be independent. **p<0.01. *p<0.05.

Interestingly, the protein expressions of GRP78 in these pairs of tissues corresponded with that of ApoAI (r^2^: −0.61, *p*<0.01) and A1AT (r^2^: −0.49, *p*<0.05) (Fig. 5). Meanwhile, the protein expression of ApoAI was correlated to that of A1AT (r^2^: 0.62, *p*<0.01, Fig. 4). The results indicated that these three putative biomarkers, GRP78, ApoAI and A1AT, were associated among each other in protein expression. On the contrast, GSTpi and GKN-1 seemed to be independent for protein expression.

### Immunohistochemistry

**Figure 6:**
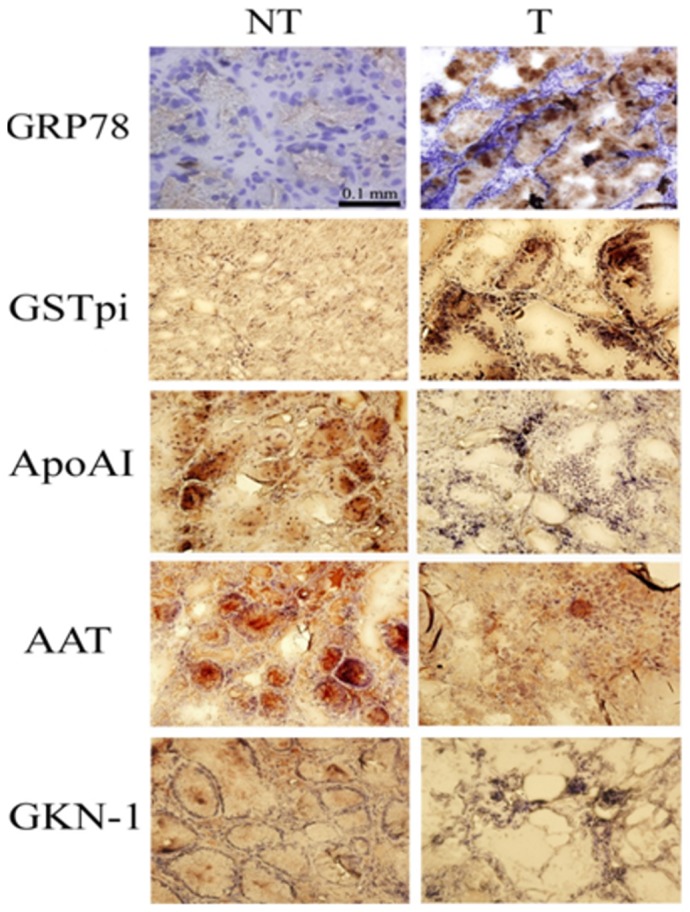
Immunohistochemistric stains were used to identify the location of the putative proteins. The up-regulated proteins, GRP78 and GSTpi, were specifically located on the gastric adenocarcinoma cells. Contrarily, the down-regulated proteins including ApoAI and A1AT were present on the normal gastric glands. Another down-regulated protein, GKN-1, was shown to appear on the mucosa of gastric tissue.

DespiteGRP78, GSTpi and A1AT being statisticallydifferent significantly, the expressional trend of other proteins was the same as the observation in the 2D-DIGE experiment. Immunohistochemistry was used to validate the acquired results from the western blotting experiment. GRP78, GSTpi, ApoAI, GKN-1, and A1AT were validated in gastric cancer tissues and non-cancerous tissues as shown in Figure 6.The protein expressions in pairs of tissues were consistent with the observation in western blotting. Particularly, the up-regulated proteins, GRP78 and GSTpi, were specifically located on the gastric adenocarcinoma cells. On the other hand, the down-regulated proteins including ApoAI and A1AT were present on the normal gastric glands. Another down-regulated protein, GKN-1, was shown as that which appeared on the mucosa of gastric tissue.

## Discussion

It is important to have biomarkers for the diagnosis and follow-up of gastric cancer.However, carcinoembryonic antigen (CEA), carbohydrate antigen (CA19-9) and CA72-4 are not commonly used in clinical practices.The present study aimed to disclose putative biomarkers by comparing the differential proteins between matched cancer and normal tissues.Afterward, these putative biomarkers were examined in validation group.Five putative proteins, including GRP78, GSTpi, ApoAI, A1AT and GKN-1, were identified by two-dimensiondifference gel electrophoresis (2D-DIGE), andmatrix-assisted laser desorption/ionization-imaging mass spectrometry (MALDI-IMS) and fatherlyvalidated in 12 clinical patients.Among five putative proteins, GRP78 and GSTpi were significantly up-regulated and specifically enhanced in cancer cells by western blotting and immunohistochemistry.In contrast,A1AT wassignificantlydown-regulated and had positive and negative correlation with the expression of ApoAI and GRP78.

Many putative biomarkers of gastric cancer have been proposed in previous studies.High levels of GSTpi in stomach carcinomas was demonstrated [14].The expressions of GSTpi were also correlated with gastric cancer stagewhere elevated GSTpi werefound in 50% at stages I or II, and 80% at stages III or IV gastric cancer patients[15].A previous study showed that GSTpi-positive patients had less five-year disease-free survival rates (49.0%) compared to GSTpi-negative patients (75.0%), indicated GSTpi was a marker of prognostic significance[16].In the present study,over-expression of GSTpi in 75% of gastric cancer patients was also demonstrated.However, the extent of over-expression of GSTpi was not correlated with clinical stages (Fig. 4).

Over-expressionof GRP78 protein was also reported as a putative biomarker of gastrointestinal cancers.GRP78 over-expressions can be detected and related to the stage and prognosis of esophageal adenocarcinoma [17], and colorectal cancer [18]. It is one of the cellular stress response proteins that play an important role in tumor biology such as regulation of apoptosis and maintaining the intracellular calcium balance [19], [20]. It was also reported that over-expressions of GRP78 by immunohistochemistry in gastric cancer specimens and metastatic lymph nodes were inversely correlated with patient survival [21]. However, the methods used in the present study were different from Zhang's report. We explored candidate proteins by 2D-DIGE to separate differential proteins and MALDI-TOF/TOF to identify peptides in matched cancer and normal tissues.In our study, the overexpression of GRP78 was increased and had a trend of correlation with clinical stages, though no statistical significance due to the limitation of case numbers.

Down-regulation of proteins may play an important role in carcinogenesis. In the present study, three proteins, ApoAI, A1AT, and GKN-1, were down-regulated in the normal gastric tissues compared to gastric cancer tissues.The relationship of ApoAI and gastric cancer has not been reported. ApoAI is one of the major protein constituents of high-density lipoprotein cholesterol (HDL-C) and plays a prime role in maintaining cholesterol transport and atheroprotective effect of HDL-C[22].Apolipoproteins such as ApoAI may modulate lipopolysaccharide-inducedinflammatory response in sepsis [23]. In the present study, it is possible that decreased ApoAI is a reflection of chronic inflammation, which is a cause of carcinogenesis. Further study is necessary to demonstrate the linkage between decrease of ApoAI and dys-regulation of inflammation.

Although increased A1AT in initial stages and correlation with the progress of gastric cancer stages was observed by Bernacka K. et al.[24], limited data could elucidate increased A1AT as a tumor marker of gastric cancer. Instead, decreased A1AT in gastric cancer was found in our study. A1AT possesses anti-inflammatory activity in vitro and contributes to the suppression of proinflammatory cytokine synthesis such as interleukin-8, TNF-alpha, interleukin-1 beta[25]. It has been reported that host genetic factors that affect interleukin-1-beta may determine the disease phenotype of H. pylori infection[26]. It is possible that decreased A1AT inhibits proinflammatory cytokine suppression effect. [25]. In our study, the correlation between decreased A1AT and ApoA1 was presumed an indicator of chronic inflammation.

Gastrokine-1 (GKN-1), possessing some mitogenic effects on intestinal epithelial cells (IEC-6), was down-regulated in gastric cancer tissue compared to matched normal gastric mucosa in our study. Gastric mucosa restoration after injury may be hampered if GKN-1 is down-regulated[27], [28].It has been reported that GKN1 is related to apoptotic signals that are important for tissue repair during neoplastic transformation[29]. The data of the present study also suggests a decreased GKN-1 can be found in gastric cancer tissues.

Proteomics-based method to identify apoptosis-related proteins in gastric cancer was previously reported by Bai Z. et al [30].Nevertheless, differential expression proteins of the present study were not identical to Bai's report suggesting ENO1, GRP78, GRP94, PPIA, PRDX1 and PTEN as potential gastric cancer biomarkers.Regarding the development of gastric cancer, it is a complicated process, and exhibits interactions between host, environment, and bacteria such as *Helicobacter pylori*.A single factor or protein could not be able to predict the occurrence and progression of gastric cancer. In the present study, five putative proteins were found to be differentially expressed in matched tissues (tumor and adjacent normal tissue). Two of them (GRP78 and GSTpi) were up-regulated and the others (ApoAI, A1AT, and GKN-1) were down-regulated. Further studies will be necessary to elucidate theseproteins asbiomarkers for detection of gastric cancer.
